# Unraveling the cytotoxicity and metabolic pathways of binary natural deep eutectic solvent systems

**DOI:** 10.1038/srep41257

**Published:** 2017-02-01

**Authors:** Yves Paul Mbous, Maan Hayyan, Won Fen Wong, Chung Yeng Looi, Mohd Ali Hashim

**Affiliations:** 1 University of Malaya Centre for Ionic Liquids (UMCiL), University of Malaya, Kuala Lumpur 50603, Malaysia; 2 Department of Chemical Engineering, University of Malaya, Kuala Lumpur 50603, Malaysia; 3 Institute of Halal Research University of Malaya (IHRUM), Academy of Islamic studies, University of Malaya, Kuala Lumpur 50603, Malaysia; 4 Department of Medical Microbiology, University of Malaya, Kuala Lumpur 50603, Malaysia; 5 Department of Pharmacology, University of Malaya, Kuala Lumpur 50603, Malaysia

## Abstract

In this study, the anticancer potential and cytotoxicity of natural deep eutectic solvents (NADESs) were assessed using HelaS3, PC3, A375, AGS, MCF-7, and WRL-68 hepatic cell lines. NADESs were prepared from choline chloride, fructose, or glucose and compared with an N,N-diethyl ethanolammonium chloride:triethylene glycol DES. The NADESs (98 ≤ EC_50_ ≥ 516 mM) were less toxic than the DES (34 ≤ EC_50_ ≥ 120 mM). The EC_50_ values of the NADESs were significantly higher than those of the aqueous solutions of their individual components but were similar to those of the aqueous solutions of combinations of their chief elements. Due to the uniqueness of these results, the possibility that NADESs could be synthesized intracellularly to counterbalance the cytotoxicity of their excess principal constituents must be entertained. However, further research is needed to explore this avenue. NADESs exerted cytotoxicity by increasing membrane porosity and redox stress. *In vivo*, they were more destructive than the DES and induced liver failure. The potential of these mixtures was evidenced by their anticancer activity and intracellular processing. This infers that they can serve as tools for increasing our understanding of cell physiology and metabolism. It is likely that we only have begun to comprehend the nature of NADESs.

The demand for “greener” solvents has led to the emergence of deep eutectic solvents (DESs). A notable feature of DESs is their melting points, which are typically lower than the freezing points of their individual components. In general, the key components of DESs include a hydrogen bond acceptor (HBA) (e.g., a salt) and hydrogen bond donor (HBD) (e.g., polyols, sugars, organic acids, amides, and amino acids, that are bonded chemically via hydrogen bonds^1^. The hydrogen bonds result from the complexation between an HBA, usually choline chloride (ChCl), and an HBD.

Several factors -such as the interactions between the salt’s anionic species and the HBD, the lattice energies of the ionic species of the DESs, the nature and asymmetry of the organic salts, and the charge delocalization that occurs through hydrogen bonding- are responsible for the significant depression in freezing points that are experienced with DESs. The freezing point depression governs the thermal stability and easy storage of DESs, because most DESs assume liquid phases between room temperature and 70 °C^2^.

In addition, other attractive properties have been associated with DESs, such as their lack of flammability, low volatility, thermal and chemical stability, tuneability, wide polarity, low vapor pressure, high solvability, and affordability (low-cost starting materials)^3^. Although they are classified in several categories (according to the chemical nature of the HBA and HBD), the most frequently encountered DESs are prepared using the quaternary ammonium salt ChCl. Because cholinium is a known component of vitamin B complex, its use for DES preparations has led to the anticipation of safe and sustainable mixtures.

DESs have many applications in various fields, such as biotransformation^4^, downstream and upstream biodiesel processes^5^,^6^, extraction of bioactive substances^7^, drug transport and solubility^8^, and nanotechnology^9^. However, research into ChCl-based DESs has not always demonstrated negligible toxicity profiles.

Following preliminary studies, Hayyan *et al*.^10^,^11^ showed that quaternary ammonium salts (ChCl) are toxic to aquatic organisms (e.g., brine shrimp), whereas phosphonium salts are toxic to aquatic and terrestrial organisms (e.g., bacteria). Subsequent studies^12^,^13^,^14^ have concluded that the inherent toxicity of DESs depends on many factors, including the molar ratio and the chemical composition of the raw materials.

Unstable molar ratios convert relatively safe mixtures into toxic eutectics. Hayyan *et al*.^11^ reported that [ChCl]-[urea/glycerol/ethylene glycol] DESs (molar ratio of 1:3) did not inhibit *E. coli* growth. Conversely, at a ratio of 1:1, these mixtures were lethal to *E. coli,* as shown by Wen *et al*.^14^. Thus, the change in ratio most likely directly increased the viscous character of these mixtures and indirectly affected their lethality.

The chemical nature of the starting materials influences the toxicity profile of the resulting DESs. In a recent study, Zhao *et al*.^15^ recorded high inhibitory values for organic acids-based DESs, in contrast to amine-/alcohol-based DESs, which were non-inhibitory. A similar observation was made by Radošević *et al*.^13^. The authors noted the formation of harmful intracellular calcium oxalate crystals following treatment of MCF-7 cells with [ChCl]-[oxalic acid] DES, whereas amine- and alcohol-based DESs were relatively non-toxic. These results support the claim that harmless DESs can be obtained using biomaterials and renewable substances.

A subset of DESs, termed natural deep eutectic solvents (NADESs), are prepared solely using raw materials of natural origin (e.g., amino acids, sugars, and organic acids). NADESs were discovered through an analysis of metabolomic data from various plant materials. Choi *et al*.^16^ argued that inexplicably high concentrations of primary metabolites in plant cells (choline, sugars, acids, amino acids, etc.) were necessary to prepare NADESs, which amount to novel cellular media (in addition to water and lipids). The purpose of these media would include the dissolution of solutes of intermediate polarity, the storage of metabolic products, cryopreservation, germination, and resistance. A separate study^17^ compiled a list of potential combinations of NADESs. Despite the lack of the physicochemical characterization of NADESs, their large-scale use requires appropriate and accurate safety policies, based on toxicology assessments. The use of ChCl as the chief component of DESs implies relatively non-toxic profiles, which have yet to be encountered *in vivo* or *in vitro*. Thus, given that most NADESs are based on ChCl, critical and in-depth analyses of their toxicity profiles are needed. Only Paiva *et al*.^18^ and Zhao *et al*.^15^ have examined the toxicity of these mixtures. Although these mixtures have demonstrated excellent viability, their intracellular mechanisms have not been examined.

In this study, the cellular impact of two NADESs that were based on ChCl and the HBDs glucose and fructose was compared to that of a DES that was based on N,N-diethylethanol ammonium chloride (DAC) and triethylene glycol (TEG) in various cancer cell lines (Fig. 1). NADESs’ cytotoxic mechanism was assessed through cellular membrane porosity and redox stress analysis. Furthermore, a novel *in vivo* cytotoxic profile of NADESs was generated. Both NADESs were prepared using a salt-to-HBD ratio of 2:1, which is optimal ratio for these mixtures^19^,^20^,^21^.

## Materials and Methods

### Chemicals and materials

N,N-diethylethanol ammonium chloride (≥98%), glucose, fructose (≥99%), and triethylene glycol (≥99%) were purchased from Merck. Choline chloride (≥97%) was obtained from Sigma-Aldrich. The human cervical cancer cell line (HelaS3), human prostate cancer cell line (PC3), human gastric cancer cell line (AGS), human skin malignant melanoma cell line (A375), human breast cancer cell line (MCF-7), and human hepatocyte cell line (WRL-68) were purchased from American Type Culture Collection (ATCC, Manassas, VA). Dulbecco’s Modified Eagle Medium (DMEM) and the Roswell Park Memorial Institute medium (RPMI 1640) were obtained from Life Technologies, Inc., Rockville, MD. Fetal bovine serum (FBS) was supplied by Sigma-Aldrich.

### Preparation of DES

Table 1 illustrates the composition, molar ratios, and symbols of the NADESs. The preparation method was similar to that reported in the literature^10^,^11^,^22^. The NADESs were prepared at a 2:1 salt-to-HBD ratio, whereas the DES followed a 1:3 molar ratio. The aqueous solutions were prepared using the same concentration of each component in the DES—dissolved in distilled water individually (Glu_aq_, Fru_aq_, ChCl_aq_, DAC_aq_, TEG_aq_) or combined (e.g., ChCl + Glu = NADES1_aq_).

### Cell culture

HelaS3, A375, AGS, and WRL-68 cell lines were grown in DMEM that was supplemented with 10% FBS, 100 U/ml penicillin, and 100 μg/ml streptomycin. MCF-7 and PC3 cell lines were grown in RPMI, supplemented with 10% FBS, 100 U/ml penicillin, and 100 μg/ml streptomycin. The cells were kept in culture flasks inside an incubator that provided a humidified atmosphere of 37 °C, with 5% CO_2_. The cells were grown to 80% to 90% confluence, as necessary for the viability assay.

### MTT viability assay

MTT cell viability assay was performed as described in the literature^12^. The EC_50_ values were obtained from an average of at least three independent experiments. The standard error of the mean (SEM) from the repeat experiments was used to derive the variations from the average EC_50_ values. The statistical analysis was performed using Graph Pad Prism 5. Statistical significance was defined when P ≤ 0.05.

### Membrane permeability assay

A total of 1 × 10^4^ cells per well were seeded onto a 96-well plate for 16 h. The cells were then treated with DESs at EC_50_ concentrations and incubated for another 24 h at 37 °C in 5% CO_2_. To examine plasma membrane permeability, cell permeability dye (Image-iT DEAD Green viability stain, Thermo Fisher) was added to live cells and incubated for 30 min, as described in the literature^23^. DEAD Green is an impermeant dye to healthy cells that becomes permeant when the plasma membrane of cells is compromised. The cells were washed twice with PBS before being fixed with 3.7% formaldehyde solution for 20 min and read on a fluorescent microplate reader at an excitation/emission wavelength of 488/515. The cells were kept overnight in PBS. The cells were visualized and the images were captured the following day using the Cellomics ArrayScan High content screening reader system (Thermo Scientific, PA, USA).

### Oxidative stress assay

Reactive oxygen species (ROS) assay was carried out to determine the influence of solvents on the production of ROSs in treated cells. A total of 1 × 10^4^ cells per well were seeded onto 96-well plate and incubated overnight at 37 °C in 5% CO_2_. The cells were then treated with EC_50_ concentrations of the solvents for 24 h, after which dihydroethidium (DHE) dye was added to the live culture for 30 min. The cells were fixed and washed with PBS as described previously^24^. The DHE dye probe is typically oxidized to ethidium in the presence of superoxides. The fluorescence intensity was measured on a fluorescent plate reader at an excitation wavelength of 520 nm and an emission wavelength of 620 nm. The values are expressed as means ± SD of three sets of experiments. The cells were visualized, and images were captured on the following day on a Cellomics ArrayScan HCS reader (Thermo Scientific).

### *In vivo* assessment

The acute toxicity of the compounds was evaluated using six Imprinting Control Region (ICR) mice per group at 8 to 12 weeks of age, with an average body weight of 25.6 g. The mice were assigned equally into 4 groups: vehicle (dH_2_O), high-dose (20 g/kg), medium-dose (10 g/kg), and low-dose (5 g/kg). Prior to administration, the mice were fasted (but allowed to drink water) overnight and for 3 to 4 h after administration of the compounds to eliminate any residual food in the gastrointestinal tract that might have complicated absorption of the test substance.

The mice were observed at 30 min and 2, 4, 24, and 48 h after administration for the onset of clinical or toxicological symptoms and mortality and behavioral changes following treatment. They were euthanized by CO_2_ asphyxiation on Day 15, and serum biochemical and histological (liver and kidney) parameters were measured using standard methods. The mortality of the mice were recorded within 14 days and used to calculate the LD_50_ for each compound. The sampled mice were housed in a specific pathogen-free facility at the University of Malaya. This work was designed to minimize animal suffering and the number of animals that was used. The study was approved by the Faculty of Medicine Animal Care and Use Committee (FOMIACUC, Approval No: 2014-05-07/PHAR/R/CYL) at the University of Malaya and was conducted in accordance with ARRIVE guidelines.

## Results and Discussion

### MTT viability assay

**Figure 2:**
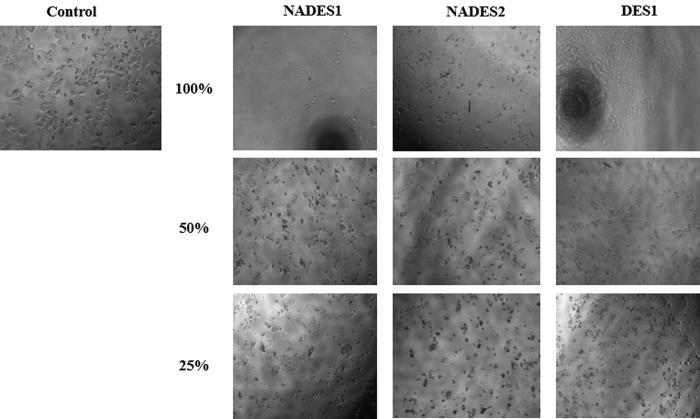
Light microscope images of MCF-7 cells submitted to NADESs/DES treatment. Control cells shown were not subjected to any treatment, and represent the 100% growth. The other cells were treated with different concentrations (100%, 50%, and 25%) of the solvents with 100% being 4.5 M.

The MTT viability assay results depict NADESs as harmful materials, based on the EC_50_ values in Table 2. A morphological assessment of HelasS3, AGS, MCF-7, and WRL-68 cell lines following NADES1 and NADES2 treatment also demonstrated the lethal effects of these mixtures. Figure 2 shows that under NADES1, NADES2, and DES1 treatment, MCF-7 cell lines shrink considerably, with their shapes and morphologies no longer consistent with viable growth. Cell confluence was significantly affected, and the culture media were filled with dead cells and those approaching the necrotic state. The same effects were observed for the other cancer cell lines (Figure S1, Supplementary Material).

The EC_50_ values for NADES1 and NADES2 ranged from 98 to 516 mM. The only other toxicological assessment of NADES ([ChCl]-[Glu]) on cancer cells had EC_50_ values ≥ 10 mM in MCF-7 cells^13^. Following the inclusion of DES1, the range of effective concentrations widened. Despite a lower endpoint, the final range of EC_50_ values (34 ≤ EC_50_ ≤ 516 mM) remained significantly higher than those reported.

Across all cells, DES1 was the most toxic mixture (Table 2). The increase in solvent toxicity followed the same sequence between all cell lines: NADES2 < NADES1 < DES1. Cell line susceptibility varied between eutectics. For NADES1, AGS cell line was the most susceptible, whereas HelaS3 cells were the least (AGS < MCF-7 < WRL-68 < A375 < PC3 < HelaS3). For NADES2, the trend was similar with one exception: WRL-86 cells were more susceptible than the breast cancer cell line (AGS < WRL-68 < MCF-7 < A375 < PC3 < HelaS3). DES1 was more toxic to the hepatic cell line than to any other cell line (WRL-68 < AGS < A375 < MCF-7 < PC3 < HelaS3). Overall, HelaS3 and PC3 cell lines were the least harmed, whereas the eutectic mixtures were increasingly lethal to AGS and WRL-68 cell lines, demonstrating that these mixtures harm normal (i.e., WRL-68) and cancerous cells, likely through a shared process in these cells. Thus, although greener than DES, NADESs can be used as potential anticancer agents but will require targeted delivery (e.g., nanoparticles) due to the risk that is posed to normal cells.

**Figure 3:**
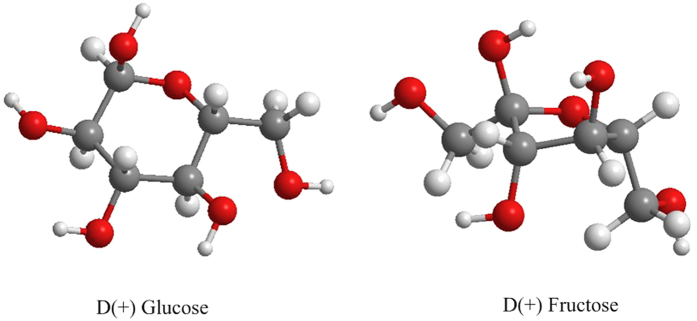
Glucose and fructose 3D models.

**Figure 4:**
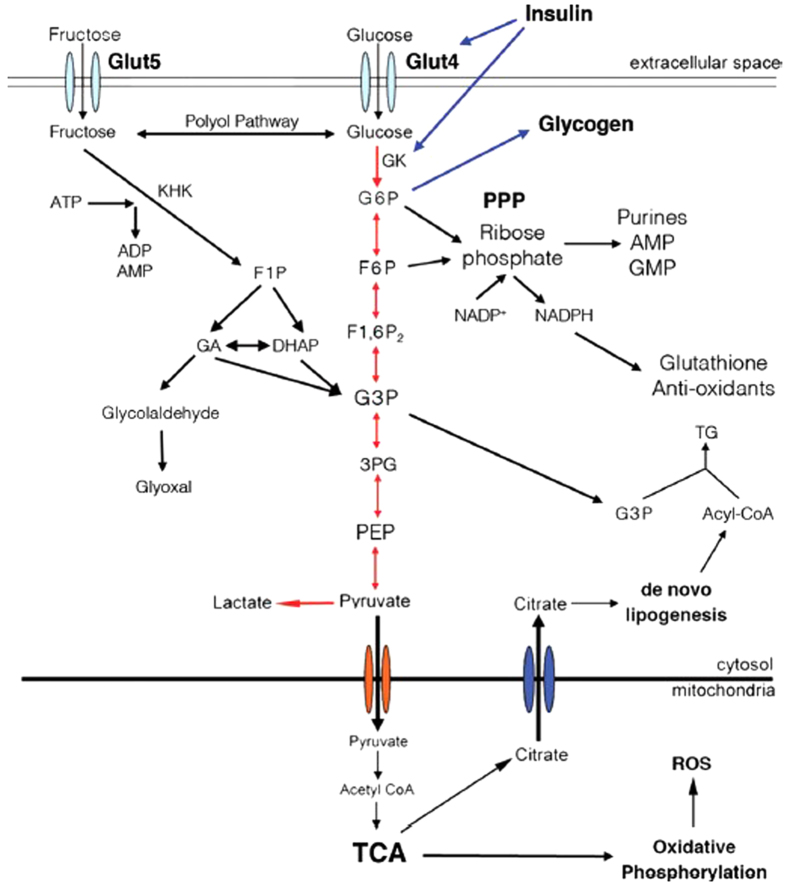
Metabolic pathway of glucose and fructose^39^. (De Gruyter *The role of fructose in metabolism and cancer*, Walter De Druyter GmbH Berlin Boston (2015). Copyright and all rights reserved, Material from this publication has been used with the permission of Walter De Gruyter Gmbh.) Glucose intracellular transport occurs by means of the insulin-dependent transporter Glut4, whereas fructose enters the cell through Glut 5. Insulin directly promote glucose metabolism through glycolysis or its storage as glycogen, by controlling the transcription of glucokinase (GK). Red arrows represent pathways used by glucose. Black arrows represent pathways used by glucose but preferentially by fructose. Metabolites and enzymes. Glut: glucose transporter; KHK: ketohexokinase; ATP: adenosine triphosphate; ADP: adenosine diphosphate; AMP: adenosine monophosphate; G6P: glucose-6-phosphate; F1P: fructose-1-phosphate; F6P: fructose-6-phosphate; F1,6P_2_: fructose1,6 biphosphate; GA: glyceraldehyde; DHAP: dihydroxyacetone phosphate; G3P: glyceraldehyde-3-phosphate; PEP: phosphoenolpyruvate; TG: triglyceride.

Figure 3 displays the chemical structures of glucose and fructose, which have the same chemical formula (C_6_H_12_O_6_). In Table 2, NADES-based glucose (NADES2) was less toxic than its fructose (NADES1) counterpart across all cell lines. The metabolic pathways that each metabolite follows upon absorption explain this difference. Glucose and fructose are reducing sugars that represent an energy source for cells. Glucose is the primary energy source for all tissues, whereas fructose is metabolized primarily by the liver. Glucose metabolism is regulated by insulin, which determines the fate of the ingested glucose. When glucose is intended to produce energy, it enters aerobic glycolysis and is phosphorylated to glucose-6-phosphate (G-6-P) by the enzyme glucokinase (GK). G-6-P can be converted to NADH, antioxidants, nucleic acids, and uric acid via the oxidative pentose phosphate pathway (PPP); to triglycerides and lipids by *de novo* lipogenesis; or to energy through the tricarboxylic acid (TCA) cycle and oxidative phosphorylation (electron transport chain) (Fig. 4).

**Figure 5:**
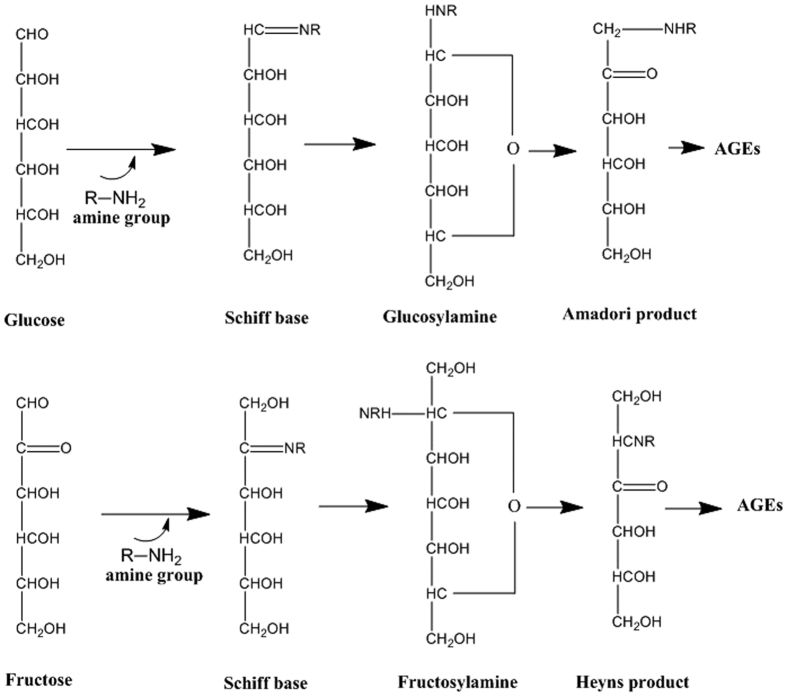
Formation of AGEs from Amadori and Heyns products of glucose and fructose, respectively.

Fructose processing is not controlled by insulin secretion; thus, fructose can nearly continuously enter the glycolytic pathway (via polyol conversion) and undergo *de novo* lipogenesis (leading to fatty acid accumulation)^25^. Under energy-deficient conditions, this pathway is an effective beneficial measure. However, at homeostasis, it can have disastrous intracellular effects. High-fructose diets increase the synthesis of advanced glycation end products (AGEs) faster than glucose-based diets. AGEs are obtained following glycation or through sugar intermediates that are produced during glycolysis. Glycation refers to the non-enzymatic modification of biomolecules (amino acids, lipids, nucleotides) following the addition of a sugar, in a process that is known as the Maillard reaction (Fig. 5)^26^. AGEs inflict serious damage to biomolecules by modifying their structure and subsequently enhancing ROS synthesis.

**Figure 6:**
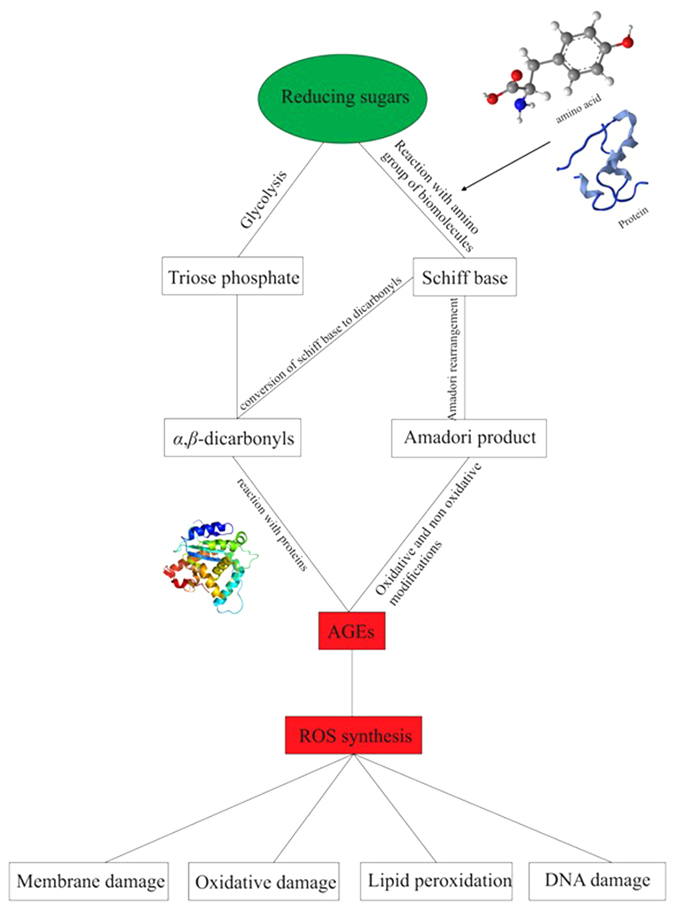
Formation of AGE and ROS from reducing sugars (fructose and glucose). Sugars use two strategies to synthesize AGEs intracellularly. The first one is through glycolysis, and the second makes use of the Maillard reaction.

Interactions of biomolecules with ROSs ultimately impair metabolic functions (Fig. 6). For instance, glycation of proteins results in biochemical dysfunction, protein crosslinks, and alteration of protein structure^27^. Membrane interactions can also be affected by the formation of lipid glycation adducts through increased membrane fluidity, the promotion of lipid peroxidation, and eventually oxidative damage^28^,^29^,^30^,^31^. Similarly, the effects of AGEs on DNA include strand breaks, unwinding of the double helix, mutations, and formation of DNA-protein and nucleotide-nucleotide crosslinks^32^,^33^,^34^. Fructose produces AGEs at least seven times faster than glucose, ultimately leading to over a 100-fold increase in ROS production compared to glucose-based AGEs. The cell is then unable to generate enough antioxidants to deal with the oxidative species^35^,^36^.

The situation is worse for cancer cells, in which upregulation of glycolysis occurs because the cells require a higher amount of carbohydrates compared with normal cells for growth, proliferation, migration, and invasion^37^. The higher carbohydrate requirement also stems from the fact that cancer cells initially use anaerobic glycolysis for glucose/fructose metabolism and thus produce less total energy (energy output is higher for aerobic glycolysis than anaerobic glycolysis). Accordingly, in cancer cells, high-glucose diets generate AGEs at a higher rate than normal cells, leading to oxidative damage, tissue damage, and inflammation, due to the imbalance between AGEs and intracellular antioxidants. As a result, glucose, despite being a desired cellular metabolite, becomes cytotoxic.

However, fructose is more cytotoxic, because the conversion and generation rates of AGEs and ROSs are significantly higher than those of glucose. Moreover, in cancer cells, fructose (converted to glucose intermediates via the polyol pathway; Fig. 4) is the preferred carbohydrate source for nucleic acid biosynthesis, whereas glucose is allocated primarily for glycolysis^38^. Fructose-based nucleic acid synthesis via the non-oxidative PPP in cancer cells stimulates an increase in uric acid synthesis^39^. Overproduction of uric acid following high-fructose diets is often associated with endothelial dysfunction, cardiovascular disease, and oxidative metabolism, with increased ROS concentrations and ultimately redox stress. This information is helpful in justifying the greater toxicity of NADES1 compared to NADES2.

Other factors, such as the physical properties of the eutectic mixtures, can be used to explain the difference in EC_50_ values. Table 3 lists the viscosity and pH of NADESs at 25 °C. Both NADESs are neutral mixtures (pH ~7), but NADES1 (11733.1 mPa.s) is more viscous than NADES2 (8045.1 mPa.s)^19^,^20^. As highly viscous materials are more toxic than less viscous compounds, it may account for the greater toxicity of NADES1.

However, in general, NADES1 and NADES2 had higher viscosity and less acidity than DES1 but were less toxic, in contrast to the greater toxicity that is expected with highly viscous mixtures. Although the natural origin of the raw materials of the NADESs is one possible reason for this phenomenon, the acidity of the DES1 appears to influence this property.

According to Table 3, NADES1 and NADES2 are neutral mixtures (pH 6.67 and 7.24, respectively). The intra-cytoplasmic pH is often found in a range that approximates that of NADES2 (7.1~7.2)^40^, which also explains why it was the least detrimental eutectic mixture. Cancer cells often have a slightly more acidic extracellular environment due to lactate and H^+^ overproduction from increased glycolysis, even in the presence of oxygen (the Warburg effect)^41^. The ensuing extracellular acidosis (5.8~7.6 in human and rodent tumors)^42^ and the resulting gradient difference can be toxic to cancer cells, which appears to be the case for NADES1 and DES1.

Riemann *et al*.^43^ confirmed that induced extracellular acidosis promotes a sustained decrease in intracellular pH and the formation of ROSs. DES1 was more detrimental than NADES1, because its pH (~1) is significantly lower, and thus, the influx of H^+^ is higher. Hence, ROSs are generated at a higher rate compared with NADES1, causing DES1 to be more toxic than NADES1. This influx of acid into the cell also explains the higher toxicity of NADES1 versus NADES2. Moreover, the gradient difference that is created by the difference in pH contributes to the cellular absorption of ions (Na^+^, K^+^, Ca^+^) and metabolites that are important for cancer progression, invasion, and migration. The influx of NADES1 and NADES2 might be relevant to the cell for specific metabolic purposes, but import of DES1 differs, because DAC and triethylene glycol metabolites are not needed as much as glucose, fructose, or ChCl. Following ingestion, TEG is metabolized by alcohol dehydrogenase to yield toxic diacid and hydroxy acid compounds, which advance metabolic acidosis^44^,^45^. Thus, their presence in high amounts promotes their cytotoxicity, rendering them lethal to the cell.

As shown in Table 2, the EC_50_ values varied between cell lines, with the lowest value associated with AGS cell line and the highest corresponding to HelaS3 cell line. These findings suggested that a gastric cancer cell line was easily affected by DESs, whereas the opposite occured for HelaS3 cell line. Given that cells are protected and compartmentalized primarily by their membranes, their interaction with NADESs must be a crucial factor for the ensuing cellular stress.

### Assessment of membrane permeability

**Figure 7:**
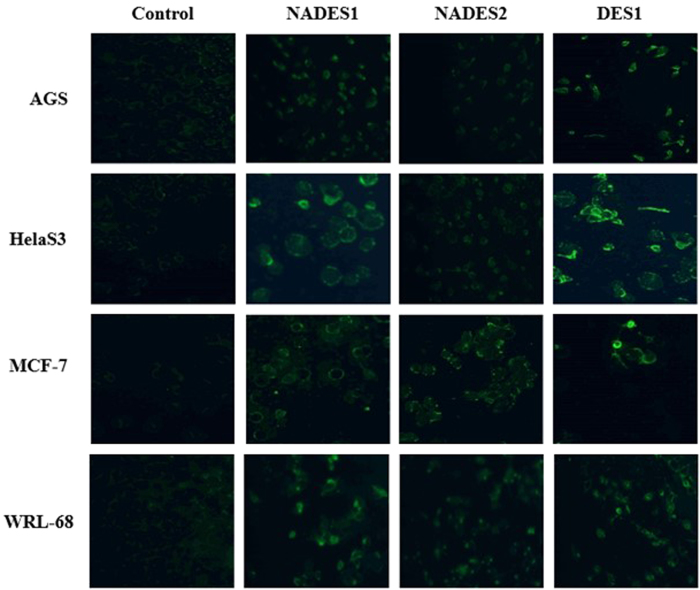
Effect of NADESs/DES on the permeability of various cell lines. Fluorescent images of AGS, HelaS3, MCF-7 and WRL-68 control and treated cells with EC_50_ concentrations of NADESs/DES.

The cell permeability assessment following NADESs treatment showed a comparable effect with the DESs that were tested by Hayyan *et al*.^12^ (Fig. 7). Using a permeability dye (Image-iT DEAD Green viability stain) as an indicator of cell membrane damage, we evaluated the severity of NADES/DES cytotoxicity. Once the dye penetrates the cytoplasm, its detection of affected or dead cells by fluorescent imaging (green) establishes the effect of the applied material.

As shown in Fig. 7, the green fluorescence of NADESs and DES is more discernible in treated cells versus the control.

In NADES-/DES-treated cells, this fluorescence stemmed from within the cells, implying increased porosity, whereas in control cells, only extracellular fluorescence of debris could be seen, suggesting that the dye did not penetrate the cells, allowing them to remain healthy. These results established the capacity of NADESs to perforate cellular membranes. However, DESs were more destructive than NADESs, based on the stronger emitted fluorescence of DES1-treated cells. The increased porosity in DES1-treated cells versus NADES1 and NADES2 might be related to the complex interactions between these mixtures and the cell membrane. The fluorescence values of each cell line before and after treatment are shown in Figure S2 (Supplementary Material).

Membranes are composed of a matrix of lipids and proteins, the structures of which regulate their permeability. The composition of membrane lipid moieties depends on the cell type and its physiological requirements and functions^46^. One of the most important constituent of lipid bilayers are phospholipids, the distribution of which creates a unique membrane potential that regulates membrane permeability and the diffusion of ionic and molecular species (due to the nature of their various head groups and phosphate moiety densities).

**Figure 8:**
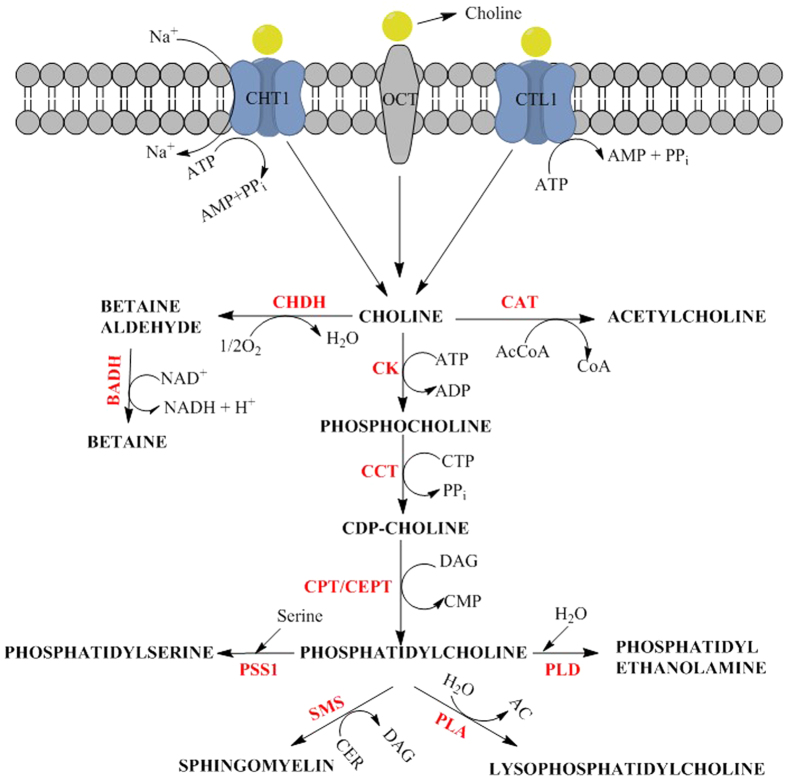
ChCl intracellular transport and metabolism. Choline enters the cell either using low-affinity facilitated diffusion via OCT transporters; high affinity Na^+^-dependent transport via CHT1 transporters; or intermediate affinity Na^+^-independent transport via CTL1 transporters. Enzymes and metabolites. CHDH: choline dehydrogenase; CAT: choline acetyltransferase; AcCoA: acetyl-coenzyme A; CoA: coenzyme A; BADH: betaine aldehyde dehydrogenase; NAD^+^: nicotinamide adenine nucleotide; NADH: reduced nicotinamide adenine nucleotide; CK: choline kinase; CCT: citydylyltransferase; CTP: cytidine triphosphate; CDP: cytidine diphosphate; CPT: cytidine cholinephosphotransferase; CEPT: choline/ethanolaminephosphotransferase; DAG: diacylglycerol; CMP: cytidine monophosphate; PSS1: Phosphatidylserine synthase 1; PLD: Phospholipase D; SMS: sphingomyeline synthase; CER: ceramide; PLA: phospholipase A; AC: acyl chains; PPi: pyrophosphate.

Phosphatidylcholine (choline head group) is the most abundant phospholipid in plasma membranes^47^. It is synthesized from choline, primarily by exogenous routes to minimize energy expenditure. Choline is pivotal in the metabolism of phosphatidylcholine and other bilayer phospholipids, such as phosphatidylserine, phosphatidylethanolamine, and sphingomyelin (Fig. 8). Thus, for normal and cancer cell lines, intracellular choline import is a crucial feature of their metabolism. Several protein families that are embedded in the membrane bilayer participate in the intracellular diffusion of choline. They consist of the polyspecific organic cation transporter (OCT) family for low-affinity facilitated diffusion, the choline transporter (CHT1) family for high-affinity Na^+^ -dependent transport; and the choline transporter-like (CTL1) family for intermediate-affinity Na^+^ -independent transport^48^,^49^. Thus, similar to glucose and fructose moieties, the diffusion of choline moieties is assisted by membrane transporters. Consequently, the entry of choline or choline-based moieties is a natural phenomenon in eukaryotic cells.

The increased permeability of NADESs-treated cells could be an indirect consequence of the limiting uptake rate of choline species through embedded membrane transport proteins and/or the threshold concentrations of these mixtures—i.e., as the rate of choline transport in cells becomes limited^50^, extracellular moieties might begin to accumulate on the cell surface, thereby increasing porosity. In contrast to NADESs, DES1 species (i.e., DAC and TEG) are not indigenous to the cell or perhaps are not required in similar amounts. Hence, their intercellular diffusion is restricted. As a result, they are instead primarily retained by the cell membrane, having a more pronounced deleterious effect. Previous studies established that the accumulation of ammonium cations (at a specific threshold concentration) on cellular membranes disrupts the lipid bilayer and induces cell death^51^. The attractions and interactions of lipid bilayers with ionic species depend on the lipid organization and density charge, the latter of which is ultimately derived from the concentrations of acidic and basic functional groups and their average association constants with hydrogen or hydroxyl ions (all of which vary between cells)^52^. For instance, the colorectal cancer cell line is associated with an overall increase in the concentration of all phospholipid types at the cell membrane, including phosphatidylinositol, phosphatidylserine, phosphatidylethanolamine, and phosphatidylcholine^53^. This property might account for the difference in EC_50_ values between cancer cell lines and the normal cell line (WRL-68).

These inferences are subject to the transport modes and import of NADESs into the cell. It is pivotal to ascertain whether the cellular entry of NADESs occurs as dissociated components or as a single molecule. This mechanism can be determined by analyzing the cytotoxic values of aqueous solutions of the raw materials of NADESs and comparing them with the EC_50_ of the pure mixtures.

In Table 4, the EC_50_ values of the aqueous solutions of NADESs and their chief materials are presented. NADES1_aq_, (101 mM) and NADES2_aq_ (145 mM) were the least toxic mixtures, followed by glucose_aq_ (58 mM), fructose_aq_ (49 mM), DES1_aq_ (54 mM), ChCl_aq_ (35 mM), DAC_aq_ (37 mM), and TEG_aq_ (13 mM). The EC_50_ values of pure NADES1 (98 mM), NADES2 (138 mM), and DES1 (46 mM) were in a similar range as those of their aqueous solutions (NADES1_aq_ −101 mM and NADES2_aq_ −145 mM). It is interesting to note that pure NADESs had significantly lower cytotoxic values than aqueous solutions of their individual elements (55 ≥ Glucose_aq_ ≤ 61 mM; 43 ≥ Fructose_aq_ ≤ 55 mM; 30 ≥ ChCl_aq_ ≤ 40 mM), suggesting that NADESs did not dissociate after crossing the cellular membrane, in contrast to the assumptions of partial or complete dissociation in the literature.

Based on the intersection between the ranges of EC_50_ values of pure NADESs (90.9 ≥ NADES1 ≤ 105.1 mM; 124 ≥ NADES2 ≤ 152 mM) and their aqueous solutions (92 ≥ NADES1_aq_ ≤ 110 mM; 138 ≥ NADES2_aq_ ≤ 152 mM), we propose that following cellular adsorption, the individual aqueous chief components of DESs form pure NADES mixtures intracellularly to reduce their cytotoxic effects on the cells. Although the dilution and solubility factors of the chief ingredients in aqueous solution as well as the type of cancer cell used (in this work, AGS) might have played a significant role, the previous suggestion would help explain the different profiles between pure NADESs and individual ingredients’ aqueous solutions the similarity between pure mixtures and their aqueous solutions. This hypothesis would somewhat be consistent with Choi *et al*.^16^ concerning the synthesis of NADESs by plant cells, except that we extrapolate it to mammalian cells. This assumption is based on the premise that if dissociation had occurred prior to absorption, then aqueous solutions of the individual raw materials would dictate the threshold concentrations at which cell death was induced. However, the pure solutions of NADESs had a distinct profile compared with aqueous solutions of their individual elements.

With regard to DES1, the ranges of its aqueous solutions (51.7 ≥ DES1_aq_ ≤ 56.3 mM) and pure mixtures (45.25 ≥ DES1 ≤ 46.75 mM) do not intersect. Moreover, the range of pure DES1 mixtures (45.25 ≥ DES1 ≤ 46.75 mM) approximates that of the aqueous solution of DAC (29 ≥ DAC_aq_ ≤ 45 mM), suggesting that DES1 dissociates in the cellular media prior to entry. The addition of water to the combination of ingredients merely reduced the toxicity of DAC, which was already determining the toxicity of the overall mixture. It can also be argued that the low EC_50_ values of DES1 are attributed to the lower requirements of DES1 species in AGS cells.

### Assessment of redox stress

**Figure 9:**
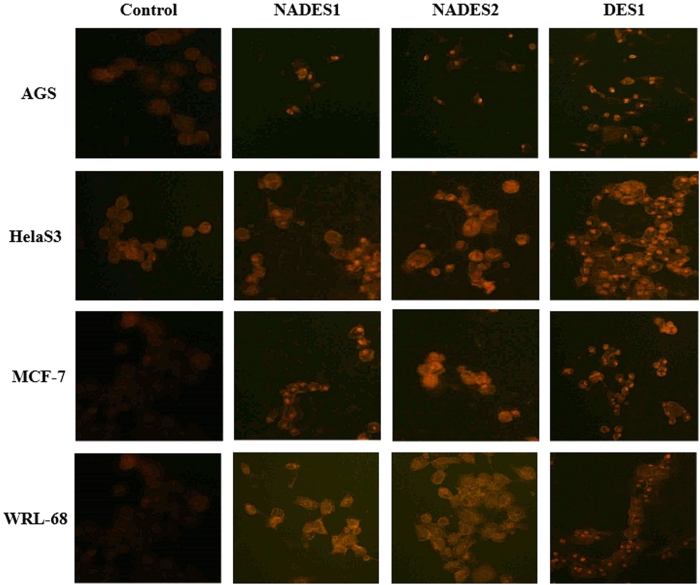
ROS production following NADESs/DES treatment. The cells were treated at each and every time with the corresponding EC_50_ values of the respective cells listed in Table 2.

The results in this study present NADESs as architects of redox stress. Figure 9 shows the images of four treated cell lines (HelaS3, AGS, MCF-7, and WRL-68) that were stained with dihydroethidium (DHE) dye. DHE freely permeates cell membranes and is thus used to monitor superoxide production. DHE reacts with superoxide ions to form a red fluorescent product (ethidium) that intercalates into DNA (forming small red dots in the cell). Although recent studies have suggested that the end product is 2-hydroxyethidium, the result is that DHE is retained well by cells and helps detect superoxide radicals^54^. The oxidized form of DHE gives off red fluorescence upon DNA intercalation. As shown in Fig. 9, the distinction between control and treated cells is notable, wherein the accentuated red fluorescence is visible among NADES-/DES-treated cells. Further, the intercalated DNA-DHE was strongly visible in NADES-/DES-treated cells.

As expected, the fluorescence was more pronounced in cells that were treated with DES1 compared with NADESs, suggesting that DES1 stimulates the synthesis of ROS to a greater extent than NADESs (correlating with the hypothesis that NADESs are easier to deal with intracellularly) or that redox stress is merely one of the mechanisms by which eutectics induce toxicity, with NADESs operating through a disparate mode of action. Regardless of which hypothesis is correct, these NADESs are less strenuous on the cell than DESs.

### *In vivo* analysis

*In vivo* profiling of NADES toxicity was performed in mice, and biochemical variables were assessed in the liver and kidneys. Table 5 shows the recorded LD_50_ values. With regard to the discussion on EC_50_ above, the increasing trend of solvent toxicity in the cell lines was as follows: NADES2 < NADES1 < DES1. During the *in vivo* assessment, this trend was reversed (DES1 < NADES1 < NADES2). This difference was attributed to the viscosity of these solvents, because NADESs were more viscous than the DES, and therefore were difficult to handle and might have failed to circulate properly in mice. This may have blocked and halted blood flow. The injected solvents were not diluted with water; thus, they remained just as viscous as during their preparation. Dilution with water alters the physicochemical profiles of DESs^55^, and can provide more manageable fluids, in terms of application and cytotoxic liability.

As seen in the blood biochemical analysis in Table 6, the eutectics preferentially targeted the liver, which is the primary organ that metabolizes fructose and glucose. Thus, it is understandable that excess levels of these reducing sugars primarily injured hepatic cells. As shown in Table 6, the parameters that varied the most were all affiliated with the liver: albumin, alkaline phosphatase (ALP), aspartate transaminase (AST), and G-glutamyl transferase. The fluctuations in the concentrations of these molecules are indicative of hepatocellular injury. Notably, the serum AST levels were 363 and 282 IU/L in NADES1- and NADES2-treated animals. The AST readings were approximately 10-fold higher than the normal range (15–37 IU/L). No increase in serum alanine aminotransferase (ALT) levels was observed. The AST/ALT ratios were greater than 5:1, implying that there was muscle or heart failure in addition to liver damage.

## Conclusion

DESs have proven in many cases to be superior to organic solvents and ionic liquids with regard to their low toxicity. This continues to be the case for NADESs which as a subset of DESs offer particularly interesting cytotoxic profiles that hint at their potential as anticancer agents. In this study, across all cell lines, NADESs were less toxic than the DES, most likely due to the nature of their raw materials. Based on their high cellular tolerance, the intracellular synthesis of NADESs must be examined thoroughly to understand their functions. In terms of mechanism, the increasing influx of oxidant species does not appear to be the sole pathway by which NADESs effect cellular necrosis. Nevertheless, *in vivo*, the higher toxicity of NADESs relative to the DES is attributed to their viscosity and overall threshold concentrations, which are often lethal. Designing materials with lower viscosity might help mitigate this issue. Further studies are needed to understanding this process in depth and could lead to the selection and design of less toxic mixtures in which can be used as a potential tool for drug delivery systems.

## Additional Information

**How to cite this article:** Mbous, Y. P. *et al*. Unraveling the cytotoxicity and metabolic pathways of binary natural deep eutectic solvent systems. *Sci. Rep.* **7**, 41257; doi: 10.1038/srep41257 (2017).

**Publisher's note:** Springer Nature remains neutral with regard to jurisdictional claims in published maps and institutional affiliations.

## Supplementary Material

Supplementary Figures

## Figures and Tables

**Figure 1:**
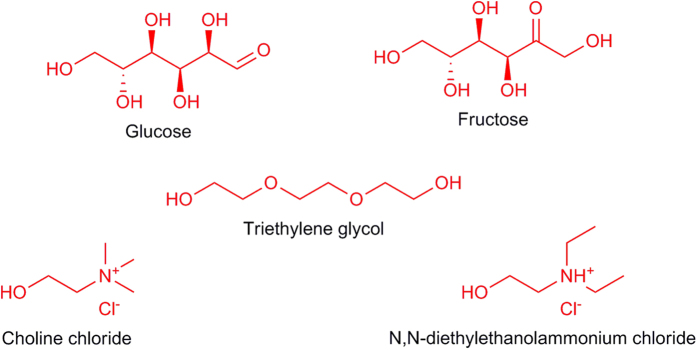
Chemical structures of the individual components of the understudied NADESs and DES.

**Table 1:** Composition, molar ratio and appearance of the understudied DESs.

NADES	Salt	HBD	Molar ratio (Salt/HBD)	Appearance
**NADES1**	ChCl	Fru	2:1	Moderately viscous liquid
**NADES2**	ChCl	Glu	2:1	Moderately viscous liquid
**DES1**	DAC	TEG	1:3	Lightly viscous liquid

**Table 2:** EC_50_ values of the understudied NADESs/DES across various cell lines.

Solvent	EC_50_ (mM)					
	Hela S3	PC3	MCF-7	A375	AGS	WRL-68
**NADES1**	436 ± 15	197 ± 17	102 ± 15	150 ± 28	98 ± 7.1	112 ± 7.8
**NADES2**	516 ± 40	340 ± 16	247 ± 8.1	265 ± 10	138 ± 14	185 ± 31
**DES1**	120 ± 15	116 ± 12	93 ± 18	91 ± 8.7	46 ± 0.75	34 ± 5.2

**Table 3:** Viscosity and pH of DES/NADESs at 25 °C^19^,^20^.

Solvent	Viscosity (mPa.s)	pH
**NADES1**	11733.1	6.65
**NADES2**	8045.1	7.24

**Table 4:** EC_50_ values of aqueous solutions of NADESs/DES and their raw materials.

Solvents	EC_50_ in AGS cell line (mM)
**Fructose**_**aq**_	49 ± 6
**Glucose**_**aq**_	58 ± 3
**ChCl**_**aq**_	35 ± 5
**DAC**_**aq**_	37 ± 8
**TEG**_**aq**_	13 ± 1.8
**NADES1**_**aq**_	101 ± 9
**NADES2**_**aq**_	145 ± 7
**DES1**_**aq**_	54 ± 2.3

**Table 5:** LD_50_ values collected upon treatment of mice with the understudied NADESs/DES.

Solvent	LD_50_ (g/mL)
**NADES1**	1.84
**NADES2**	1.24
**DES1**	4.46

**Table 6:** Biochemical analysis of blood following NADESs/DES treatment.

Clinical Chemistry	NADES1	NADES2	DES1	Unit	Ref. Range	
**Renal Function Test**						
Sodium	146	152 ± 5	150 ± 7	mmol/L	136	145
Potassium	9.2	9.2 ± 1.4	8.7 ± 0.7	mmol/L	3.6	5.2
Chloride	105	111 ± 7	110 ± 8	mmol/L	100	108
Carbon dioxide	26.8	22.1 ± 4	18.7 ± 4.3	mmol/L	21	30
Anion Gap	23	28 ± 1	27 ± 3	mmol/L	10	20
Urea	9.7	9.0 ± 2	8.9 ± 1.1	mmol/L	2.5	6.4
Creatinine	—	18	11	umol/L		
**Liver Function Test**						
Total Protein	53	52 ± 9	51 ± 3	g/L	64	82
Albumin	14	13 ± 4	13	g/L	35	50
Globulin	39	39 ± 5	38 ± 3	g/L	23	35
Total Bilirubin	2	3	2 ± 2	umol/L	3	17
Conjugated Bilirubin	<1	1	<1	umol/L	0	3
Alkaline Phosphatase (ALP)	34	37 ± 14	30 ± 19	IU/L	50	136
Alanine Aminotransferase (ALT)	70	45 ± 4	54 ± 27	IU/L	12	78
Aspartate transaminase (AST)	363	282 ± 18	349 ± 23	IU/L	15	37
G-Glutamyl Transferase	<3	<3	<3	IU/L	15	85
**Lipid Profile**						
Triglyceride	1.2	1.1 ± 0.6	0.7 ± 1.1	mmol/L	<1.7	—
Total Cholesterol	2.0	3.1 ± .06	2.3 ± 0.9	mmol/L	<5.2	—
HDL Cholesterol	2.01	2.85 ± 0.57	2.24 ± 0.79	mmol/L	<1.1	—
