# Supplementary material for: Unraveling the cytotoxicity and metabolic pathways of binary natural deep eutectic solvent systems
Source: Sci Rep. 2017 Feb 1;7:41257. doi: 10.1038/srep41257 (PMC5286504; doi:10.1038/srep41257)
Supplement: Supplementary Figures [file srep41257-s1.doc]

**Unraveling the cytotoxicity and metabolic pathways of binary natural deep eutectic solvent systems**

Yves Paul Mbous^a, b^, Maan Hayyan^a, c*^, Won Fen Wong^d^, Chung Yeng Looi^e^, Mohd Ali Hashim^a,b^

^a^University of Malaya Centre for Ionic Liquids (UMCiL), University of Malaya, Kuala Lumpur 50603, Malaysia

^b^Department of Chemical Engineering, University of Malaya, Kuala Lumpur 50603, Malaysia

^c^Institute of Halal Research University of Malaya (IHRUM), University of Malaya, Kuala Lumpur 50603, Malaysia

^d^ Department of Medical Microbiology, University of Malaya, Kuala Lumpur 50603, Malaysia

^e^Department of Pharmacology, University of Malaya, Kuala Lumpur 50603, Malaysia

^*^E-mail: maan_hayyan@yahoo.com, maan.hayyan@gmail.com; Tel/Fax No: +6-03-7967-5311


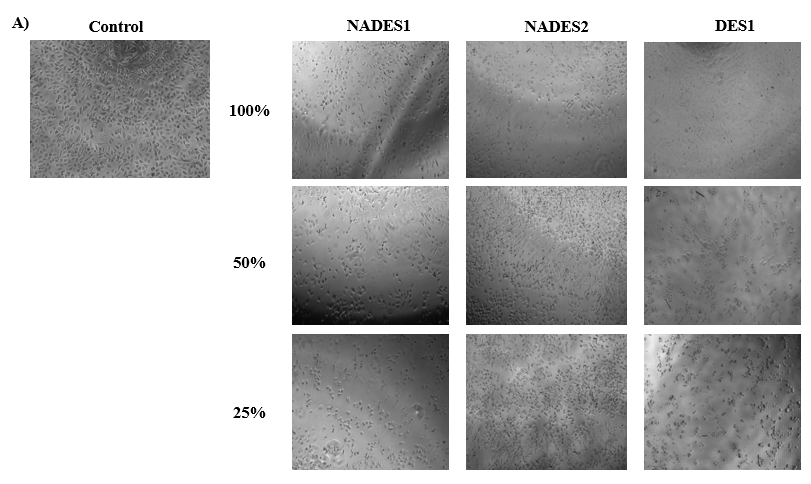


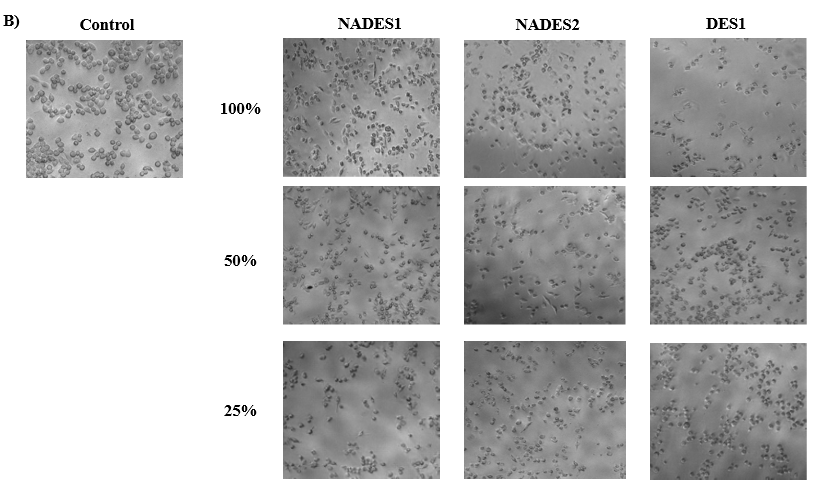


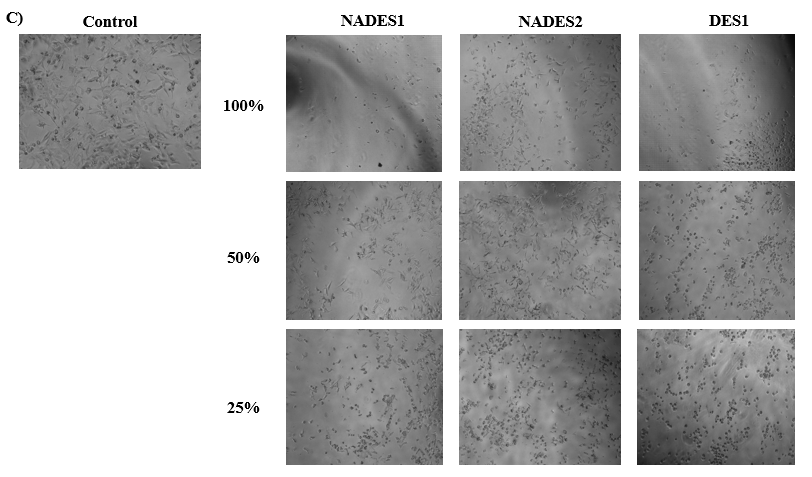


**Figure S1: Light microscope images of A) AGS, B) HelaS3, and C) WRL-68 cell lines submitted to NADESs/DES treatment.** Control cells shown were not subjected to any treatment, and represent the 100% growth. The other cells were treated with different concentrations (100%, 50%, and 25%) of the solvents with 100% being 4.5 M.


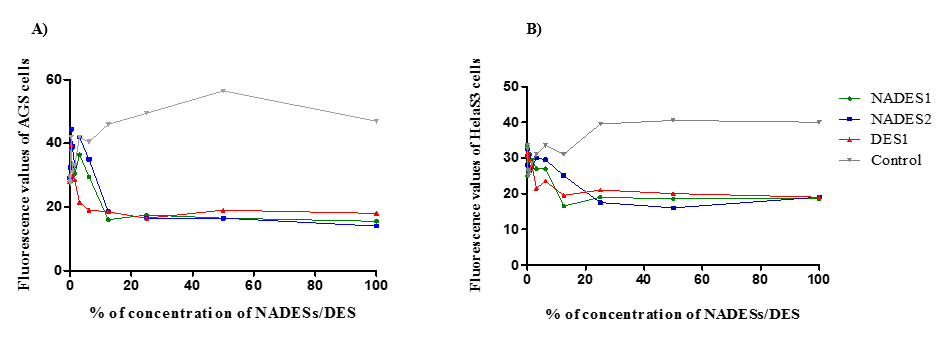


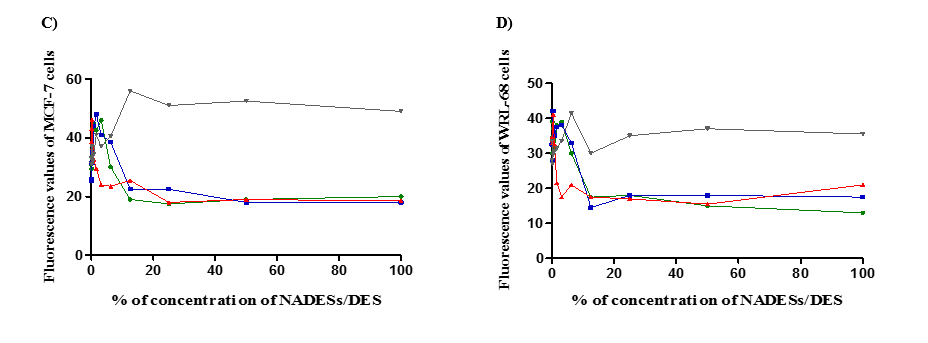


**Figure S2: Fluorescence emission from A) AGS, B) HelaS3, C) MCF-7, and D) WRL-68 cell lines over different NADESs/DES concentrations.** The fluorescence of control cells (although not subjected to any treatment) is as expected higher than those of the treated cells (given the higher amount of the former). At 40% of NADESs/DES concentration, the fluorescence intensity emitted by all four types of cells is similar. At that particular concentration, most cells are dead or at least gravely affected by the solvent.
